# Gender equity in vision care seeking behavior among caregivers: evidence from a randomized controlled trial in rural China

**DOI:** 10.1186/s12939-022-01625-4

**Published:** 2022-02-19

**Authors:** Huan Wang, Claire Cousineau, Yingjie Fan, Sarah-Eve Dill, Matthew Boswell, Scott Rozelle, Xiaochen Ma

**Affiliations:** 1 Stanford Center on China’s Economy and Institutions (SCCEI), Freeman Spogli Institute for International Studies, Stanford University, Stanford, California 94305-6055 USA; 2 China Center for Health Development Studies, Peking University, 112 Shu Wahh Building, 38 XueYuan Road, Haidian District, Beijing, 100191 China

**Keywords:** Gender equity, Healthcare seeking behavior, Rural China, Randomized controlled

## Abstract

**Background:**

Despite rising incomes and rapid economic growth, there remains a significant gender gap in health outcomes among rural children in China. This study examines whether the gender gap in child health is related to the behavior of caregivers when seeking healthcare, and whether healthcare subsidies help to bridge the gender gap in rural health outcomes.

**Methods:**

Focusing on vision care specifically, we draw on data from a randomized controlled trial of 13,100 children in Gansu and Shaanxi provinces in China that provided subsidized eyeglasses to myopic children in one set of schools (henceforth, referred to as the *treatment schools*) and provided prescription information but not subsidized eyeglasses to myopic children in another set of schools (*control schools*).

**Results:**

The baseline results reveal that while female students generally have worse vision than male students, they are significantly less likely than male students to be taken by their caregivers to a vision exam. The experimental results indicate, however, that caregivers respond positively to both health information and subsidized healthcare, regardless of the gender of their children. When prescription information is paired with a subsidy voucher for healthcare (a free pair of eyeglasses), the uptake rate rises dramatically.

**Conclusions:**

The gender gap in healthcare can be minimized by implementing subsidized healthcare policies.

**Trial registration:**

The protocol for this study was approved in full by Institutional Review Boards at Stanford University (Palo Alto, California, USA) and the Zhongshan Ophthalmic Center of Sun Yat-sen University (ZOC, Guangzhou, China). Permission was received from local Boards of Education in each region and from the principals of all schools. The principles of the Declaration of Helsinki were followed throughout. The original trial (Registration site: http://isrctn.org. Registration number: ISRCTN03252665) was designed to study the effect of providing free spectacles on children’s educational performance. The original trial was retrospectively registered on 09/25/2012.

**Supplementary Information:**

The online version contains supplementary material available at 10.1186/s12939-022-01625-4.

## Introduction

The plight of girls in low-income countries—in terms of both health and education—has drawn attention from researchers in a variety of fields. Empirical evidence has shown that female children in low-income countries often have worse health outcomes than their male counterparts [1–9]. Specifically, in China, there is extensive literature documenting the wide gender gap in health and education in the decades following the founding of the People’s Republic in 1949 [10–13]. However, in recent years, China’s government has taken steps to address these disparities. More than three decades of economic reforms starting in 1980 have lifted China from a low-income country to a middle-income country [14]. Alongside its economic growth, China’s government has carried out a series of reforms to improve the quality of education and health care for children in the past decade. Prominent examples of these reforms include the elimination of tuition and fees for public school through grade nine, subsidized school meal programs in impoverished counties, and a single payer national health insurance scheme for rural residents [1, 15, 16].

However, evidence suggests that gender disparities in health outcomes remain, particularly among rural children. Girls in rural China tend to have worse general physical health than boys, as well as suffer disproportionately more than boys from specific health issues, such as anemia [17], malnutrition [18] and being underweight [19–22]. These recent studies reveal stubbornly low health outcomes for girls when compared to boys and may conflict with the hypothesized narrowing of the gender gap, at least as it relates to health.

A series of studies of have suggested that one reason for the worse health outcomes of girls in rural China may be due to gendered differences in healthcare seeking behaviors by their caregivers [12, 23–29]. There is a strong literature base showing that rural China has traditionally valued sons over daughters [12, 23–25]. To the extent that this still persists, caregivers may be more likely to allocate limited resources to more valued children—in this case, sons [25]. Studies have shown that girls who are left-behind children and girls from families with low socioeconomic status tend to have worse health outcomes, since families with limited money or time may be more likely to exhibit gender bias [26–29]. Given these potential factors, families in rural China might behave differently in seeking healthcare for female and male children, resulting in a sustained gender gap. Because it is particularly challenging to draw causational inferences regarding gender on child health outcomes, to the best of our knowledge, nearly all available research is based on an observation design. This makes it difficult to identify whether gendered healthcare seeking behavior is the cause of the gender gap in health outcomes in rural China.

In addressing healthcare outcomes and examining gendered healthcare seeking behavior, refractive error among children in rural China is of particular concern: the prevalence of refractive error is one of the highest in the world, yet many rural students with myopia do not have glasses [30–32]. A recent study conducted in the same area as our study shows that about 25% of students in grades 4 and 5 have myopia [33]. However, recent investigations in rural China have found that fewer than one third of children who need glasses own them, and even fewer actually wear them [34]. According to Yi et al. (2015), more than 85% of children in rural China with myopia do not wear glasses [33]. While these studies offer concerning statistics regarding myopia in rural China generally, they do not specifically examine whether a gender gap exists when addressing myopia among children.

Several factors contribute to the high rates of uncorrected vision problems found in these studies, namely limited awareness of vision problems, barriers to accessing vision care, and an absence of affordable eyeglasses. Research suggests that a lack of awareness about vision care contribute to low usage rates in China [34]. For example, a large number of myopic students in rural China do not realize that they have vision problems [33, 34]. Additionally, rural students and their families may not know how to address vision problems, or they may not have access to vision screenings in the area they live [33]. Moreover, the costs associated with acquiring eyeglasses can be substantial for rural families. On top of travel costs from distant villages to the county seat (where most vision care centers are located), the average cost for a pair of glasses is around RMB 376 [35]. According to the National Statistics Bureau, the annual income per capita in rural China in 2016 was RMB 12,363, which means that a pair of glasses around a third (36.5%) of the average monthly income for rural households. For these reasons, vision care remains limited in rural China [36]. Therefore, a well-run government program providing subsidized vision care services for rural youth may be needed.

The high prevalence of myopia among children in rural China lends itself to examining the gender gap in child health outcomes and healthcare interventions. More specifically, if the gender gap in child health outcomes is indeed due to differences in the healthcare seeking behavior of caregivers, another question that arises is whether healthcare interventions may reduce gender bias in healthcare seeking behavior. Considering that resource limitations may be driving the gender gap (as discussed above), it is possible that interventions subsidizing healthcare may encourage caregivers to seek healthcare at equal rates for both male and female children. However, to the best of our knowledge, no studies have examined whether or how healthcare interventions may differently affect healthcare seeking behaviors for caregivers of girls and boys.

The present study aims to fill these gaps in the literature by examining gendered differences in healthcare seeking behaviors among caregivers of children with myopia in rural China. Specifically, we pursue three objectives. First, we examine whether gender differences still exist in health outcomes and healthcare seeking behaviors in rural China. We examine gender differences for the full sample, as well as among subgroups based on household income and parental migration status. Second, we estimate the average impact of subsidized healthcare vouchers on student healthcare uptake and usage. Finally, we explore how families with male and female children respond to healthcare interventions, both for the full sample and across subgroups.

To meet these objectives, this study draws on data from an in-the-field randomized controlled experiment of an intervention providing eyeglasses prescriptions and vouchers for subsidized eyeglasses to myopic children. The main results of the study are reported by Ma et al. (2014), who examined program impacts on eyeglasses uptake, eyeglasses usage, and student academic performance [32, 37]. Providing prescription information and subsidized eyeglasses to myopic children is a useful setting to study caregivers differential healthcare seeking behaviors. Although myopia treatment can yield important gains in children’s wellbeing and productivity, it is a health condition that is not acute and has few obvious symptoms. For such health conditions, preemption on the part of caregivers may be a key factor in uptake of remediation [38, 39]. This experiment therefore allows us to examine whether rural families might behave differently in seeking proactive healthcare remediation for female and male children, rather than seeking healthcare in response to children’s obvious symptoms.

The remainder of the paper is organized as follows. Section 2 describes the experiment and data collection. Section 3 presents the results. Section 4 discusses the policy implications of the results. Section 5 concludes. Section 6 provides a list of abbreviations.

## Methods

### Study setting and sampling technique

This study is based on an experiment that took place in two adjacent provinces of western China: Shaanxi and Gansu. Economically, Shaanxi represents an average province in China. According to the Chinese National Statistics Yearbook, the per capita Gross Domestic Product (GDP) of Shaanxi in 2012 (USD 6108) was ranked 14th out of China’s 31 provincial administrative regions and was similar to the national average for the same year (USD 6091). In contrast, Gansu is more typical of poor rural areas in China. The GDP per capita of Gansu was USD 3100 in 2012, making Gansu the second-poorest province in the country [40]. The experiment was implemented in one prefecture, containing seven to 10 counties, in each of the two provinces. The two sample prefectures are similar to their respective provinces in terms of their economic conditions; therefore, our sample provinces and prefectures can be considered representative of both average and below-average socioeconomic circumstances in rural China. Given the fact that our focus is on gender difference in rural China, these two study areas allow us to examine how differences in socioeconomic status may affect healthcare seeking behaviors for caregivers of boys and girls.

To implement the study plan, the research team followed a three-step sampling protocol. First, 167 townships were randomly drawn from the two prefectures. Second, one school per township was randomly selected for inclusion in this experiment to minimize the possibility of inter-school contamination. Third, within each school, the researchers selected one grade four class and one grade five class. Fourth and fifth grade students were chosen for this sample because previous studies in rural China have found that the onset of myopia typically begins around age 8 to 10, which coincides with fourth and fifth grade in rural Chinese schools [41]. All children in each sample class were surveyed and administered visual acuity examinations.

### Experimental design

Following a baseline survey and vision examinations (described below), each of the 167 schools was randomly assigned to one of two groups: Voucher or Prescription. The research team randomly assigned 83 sample schools to the Voucher group and 84 sample schools to the Prescription group. To improve power, randomization was stratified by county and by the number of children in the school found to need eyeglasses. In total, this yielded 45 strata. Our analysis takes this randomization procedure into account [42]. The trial was approved by the Stanford University Institutional Review Board (No. ISRCTN03252665, registration site: http://isrctn.org).

The two groups were designed as follows:

Voucher Group: each student diagnosed with myopia was given a voucher redeemable for one pair of free glasses at an optical store located in the county seat, as well as a letter to his/her parents informing them of their child’s prescription. In other words, this intervention included both information (the child’s prescription) and subsidized vision care (the voucher for free glasses). Vouchers were non-transferable, as they contained personal information including the student’s name, school, county, and the student’s prescription. Individuals were required to present their identification in person to redeem the voucher. Program eyeglasses were pre-stocked in the retail store of one previously chosen optometrist per county, all of which were located in the county seats. The distance between each student’s school and the county seat varied substantially within our sample, ranging from 1 km to 105 km with a mean distance of 33 km. While the eyeglasses were free, the cost of the trip in terms of time and transport were born by family of the student.

**Fig. 1:**
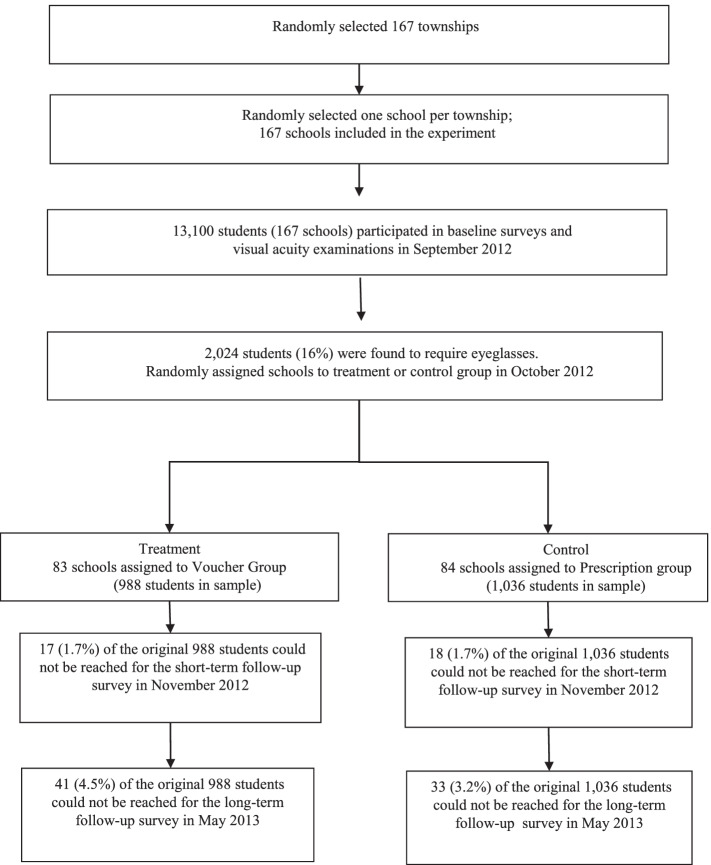
Trial Profile

Prescription Group: Myopic students in the Prescription group were given a letter to their parents informing them of their child’s myopia status and prescription. No further action was taken. Therefore, caregivers in the Prescription group only received information on their children’s myopia status. Unlike the families in the Voucher group who could redeem the voucher for one pair of free eyeglasses, the families in the Prescription group would have to purchase the eyeglasses. If they opted to purchases glasses, they would also have to travel to the county seat, as there were no optical stores located outside of the county seats in any of the sample counties. Figure 1 shows the trial profile of this study.

### Data collection

#### Baseline survey

The baseline survey for this study was conducted in September 2012 and collected comprehensive and detailed information on students, households and schools. The student-level baseline survey focused on basic demographic information of sample students, including age, gender, boarding status, and whether the student’s parents worked away from home for more than six months per year. For the purpose of our analysis, students with both parents working away from home for more than six months per year are defined as left-behind children.^1^

Household surveys were also given to all students as well as their caregivers. The head teacher of each classroom collected the completed household surveys and forwarded them to the survey team. The household survey collected information on households that children would likely have difficulty answering, such as parental education levels, whether any family members owned or wore glasses, and the value of household assets. A household asset index was calculated for each family using a list of 13 items weighted by the first principal component.^2^ In addition, information was collected on the distance between the school and the county government seat.

Additionally, the research team collected data on student vision health during the baseline survey. This included information such as whether the student’s family had ever taken him/her to a vision exam, whether the student owned eyeglasses before the baseline survey, whether the student had trouble reading blackboard in the classroom, whether the student usually blinked his/her eyes or turned his/her head to see things clearly, student knowledge of myopia,^3^ and whether the student thinks he/she is myopic.

#### Vision examination

After the baseline survey, all students were administered a two-step vision examination. In the first step, a team of two trained staff members conducted visual acuity screenings using Early Treatment Diabetic Retinopathy Study (ETDRS) eye charts, which are accepted as the worldwide standard for accurate visual acuity measurement. Students who failed the visual acuity screening test (visual acuity less than or equal to 0.5, or 20/40, in either eye)^4^ were enrolled in a second vision test that was carried out at each school one or two days after the first test.

The second vision test was conducted by a team with an optometrist, a nurse and a staff assistant and involved cycloplegic automated refraction with subjective refinement to determine prescriptions for students who failed the visual acuity screening test. Cycloplegia refers to the use of eye drops to briefly paralyze the muscles in the eye that are used to achieve focus. The procedure is commonly used during vision exams for children to prevent them from reflexively focusing their eyes and rendering the exam inaccurate.

To calculate and compare different visual acuity levels, a linear scale with constant increments is needed. In the field of ophthalmology/optometry, LogMAR is one of the most commonly used continuous scales. This scale uses the logarithm transformation: LogMAR = log10 (MAR). The variable MAR, short for Minimum Angle of Resolution, offers a relatively intuitive interpretation of visual acuity measurement—it has a constant increment of 0.1 across its scale, and each increment indicates approximately one line of visual acuity loss in the ETDRS chart. Therefore, higher LogMAR values indicate worse visual acuity. According to the results of the visual acuity examinations, more than 95% of the cases of poor vision in the sample were due to myopia. Therefore, for simplicity, the following analysis will use myopia to refer to all vision problems.

#### Eyeglasses uptake and usage

We focus our analysis on two variables: eyeglasses uptake rates and eyeglasses usage rates. The analysis is based on data collected from a short-term and a long-term follow-up survey. The short-term follow-up survey was conducted in early November 2012, one month after vouchers were distributed. The long-term follow-up survey was conducted in May 2013, seven months after vouchers were distributed.

Eyeglasses uptake in our study is defined by eyeglasses ownership. Specifically, we define uptake as a binary variable taking a value of one if a student owned a pair of eyeglasses at baseline or acquired one during the program (regardless of the source) and taking a value of zero if the student did not own or acquire glasses by the time of the short-term or long-term follow-up. Students that had been diagnosed with myopia were given an additional short questionnaire during each of the follow-up surveys that included questions about whether they owned eyeglasses and how they acquired them. If a student indicated he or she did have glasses but was not wearing them, enumerators confirmed the student’s response by asking to check the glasses. If the eyeglasses were at home, the research team followed up with phone calls to the student’s caregivers to confirm that the student actually owned a pair of eyeglasses.

Eyeglasses usage is a binary variable measuring whether a student wears eyeglasses on a daily basis. During the short- and long-term follow-up surveys, all students were asked to report whether they wore eyeglasses regularly. To reduce reporting bias, teams of two enumerators made unannounced visits to all 167 schools in advance of the long-term follow-up survey. During the unannounced visits, enumerators were given a list of the students diagnosed with myopia at baseline and recorded whether the students were observed to be wearing glasses. Student responses were then double-checked with the data collected during t unannounced visits. This process ensures that the results of our analysis using the student response data are reliable.

### Balance and attrition

Of the 13,100 students in 167 sample schools who were given vision examinations at baseline, 2024 students (16%) were found to require eyeglasses. Only these students are included in the analytical sample. Of these, 988 students in 83 sample schools were randomly assigned to the Voucher group, and 1036 students in 84 sample schools were randomly assigned to the Prescription group.

Table 1 shows the balance check of basic characteristics and vision for the two groups. The first column of Table 1 shows the mean and standard deviation in the Prescription group, while Column 2 shows the mean and standard deviation in the Voucher group. We then tested the difference between students in the Prescription and Voucher groups, adjusting for clustering at the school level (column 3). The balance check shows that there are no significant differences between the two groups at baseline in terms of student demographic characteristics or vision, indicating a consistent balance across the two experimental groups.

**Table 1:** Basic characteristics balance check across experimental groups

	Prescription Group	Voucher Group	Difference	*P*-value
Variable	(1)	(2)	(2)–(1)	
1. Age (years)	10.546	10.513	0.033	0.673
	(0.058)	(0.054)		
2. Female (1 = yes)	0.501	0.520	−0.019	0.379
	(0.016)	(0.015)		
3. Left-behind child (1 = yes)	0.100	0.124	−0.024	0.138
	(0.011)	(0.012)		
4. Boarding at school (1 = yes)	0.227	0.185	0.042	0.377
	(0.035)	(0.032)		
5. Number of siblings	1.421	1.384	0.037	0.591
	(0.052)	(0.046)		
6. Father completed high school (1 = yes)	0.157	0.134	0.024	0.229
	(0.014)	(0.014)		
7. Mother completed high school (1 = yes)	0.099	0.078	0.021	0.172
	(0.012)	(0.010)		
8. At least one family member wears glasses (1 = yes)	0.347	0.325	0.022	0.322
	(0.015)	(0.017)		
9. Household asset index	−0.053	−0.064	0.011	0.923
	(0.086)	(0.075)		
10. Distance from school to the county seat (km)	34.688	32.064	2.624	0.516
	(2.613)	(3.086)		
11. Baseline visual acuity (LogMAR)	0.647	0.621	0.027	0.104
	(0.011)	(0.012)		
12. Family has taken student to a vision exam (1 = yes)	0.302	0.353	−0.050	0.122
	(0.023)	(0.023)		
13. Student has glasses at baseline (1 = yes)	0.182	0.192	−0.010	0.670
	(0.016)	(0.017)		
14. Student can see the blackboard from his/her seat (1 = yes)	0.520	0.495	0.025	0.436
	(0.025)	(0.020)		
15. Student blinks eyes or turns head to see things clearly (1 = yes)	0.639	0.616	0.023	0.408
	(0.018)	(0.021)		
16. Myopia knowledge index (Score 0–9)	2.980	3.095	− 0.115	0.300
	(0.080)	(0.077)		
17. Student thinks he/she is myopic (1 = yes)	0.451	0.470	−0.019	0.544
	(0.024)	(0.020)		

*Notes:* Visual acuity is measured by the LogMAR of the worse eye. Higher LogMAR values indicate worse visual acuity; students with normal vision would have LogMAR value less than or equal to 0.0. The value displayed for t-tests are the differences in the means across the groups. Standard errors are clustered at school level

Since we focus our analysis on the differences in caregiver healthcare seeking behavior based on the gender of a caregiver’s child, the main variable in our baseline analysis is whether caregivers have taken their child to a vision examination. Irrespective of gender, less than 35% students with myopia had been taken to a vision examination by their caregiver prior to the study, indicating insufficient awareness of potential vision problems on the part of caregivers (Table 1, Row 12). In addition, only about 18% of students who required a pair of eyeglasses actually had them at the time of the baseline survey (Row 13). Most of the myopic students in our sample could not see the blackboard in their classrooms and would cope with vision problems by blinking their eyes or turning their heads to see more clearly (Rows 14 and 15).

The baseline data also show that rural students in the sample have a low level of awareness regarding myopia, as measured by scores on the myopia knowledge index and whether students recognize that they are myopic. The total possible score on the myopia knowledge index is 9 points. In the sample, students scored an average of about 3 points, signaling a considerable deficiency of myopia knowledge. Therefore, it is unsurprising that nearly half (47%) of the students with myopia in the sample were not aware of their myopic condition at baseline (Row 17).

Figure 1 presents the trial profile of this study. The figure shows that among the original 988 students assigned to the Voucher group, 17 of (1.7%) were no longer present to fill out the short-term follow-up survey (one month after the baseline). Similarly, in the Prescription group, 18 of the original 1036 students (1.7%) were not present to fill out the short-term follow-up survey. By the long-term follow-up survey (7 months after the baseline), 44 students in the Voucher group (4.5%) and 33 students in the prescription group (3.2%) were no longer present.

We also test for differential attrition across the Voucher and Prescription groups. To do so, we first construct indicators for attrition in the short-term or long-term (1 = attrition). We then regress different baseline covariates on a treatment indicator, the attrition indicator (for the short-term and long-term, respectively), and the interaction between the two. The results, presented in Additional file 1: Table 1, show that there are no statistically significant differences in attrition patterns between the Voucher and Prescription groups in terms of a variety of baseline covariates at the time of both the short-term and long-term follow-up surveys. The only exception is in the short term attritors in the Voucher group were less likely to believe that eyeglasses harm vision compared with attritors in the Prescription group. This difference is significant at the 5% level (Additional file 1: Table 1, row 3, column 6).

### Statistical approach

To estimate how eyeglasses uptake and usage changed for children in the Voucher group relative to children in the Prescription group, we use both unadjusted and adjusted ordinary least squares (OLS) regression models. In both models, we estimate parameters in the short-term and in the long-term. The basic specification of the unadjusted model is as follows:

1 $${\mathrm{y}}_{\mathrm{ijt}}=\upalpha +{\upbeta}_{\mathrm{V}}{\mathrm{V}\mathrm{oucher}}_{\mathrm{j}}+{\upvarepsilon}_{\mathrm{ijt}},$$

where *y*_*ijt*_ is a binary indicator for the eyeglass uptake or eyeglasses usage of student *i* in school *j* in wave *t* (short-term or long-term follow-up). *Voucher*_j_ is a dummy variable indicating schools in the Voucher group, taking a value of 1 if the student’s school was assigned to the Voucher group and 0 if the school was assigned to the Prescription only group. ε_ijt_ is a random error term.

To improve the efficiency of the estimated coefficient of interest, we also use an adjusted model with additional covariates:

2 $${\mathrm{y}}_{\mathrm{ij}\mathrm{t}}=\upalpha +{\upbeta}_{\mathrm{V}}{\mathrm{V}\mathrm{oucher}}_{\mathrm{j}}+{\mathrm{X}}_{\mathrm{ij}}+{\upvarepsilon}_{\mathrm{ij}\mathrm{t}},$$

where X_ij_, following the analytical methods of the trial conducted by Ma et al. (2014), represents a vector of baseline variables that would be correlated with vision care seeking behaviors. These baseline variables includes demographic factors (years of age, gender), socioeconomic and family factors (number of siblings, whether he/she is a left-behind child, whether he/she is boarding at school, parental education, whether a family member wears eyeglasses, household asset index, distance from school to the county seat), and vision-related factors (visual acuity, whether he/she already had eyeglasses, whether he/she could see blackboard from his/her seat, whether he/she blinks his/her eyes or turns his/her head to see thing clearly, his/her awareness and knowledge of myopia, the severity of myopia measured by LogMAR). Details of the measures of each variable are described section 3.3.1 above.

To analyze this study’s main question of interest—whether caregivers respond to the Voucher intervention differently depending on the gender of the student—we estimate parameters using the following heterogeneous effects model:

3 $${\mathrm{y}}_{\mathrm{i}\mathrm{j}\mathrm{t}}=\upalpha +{\upbeta}_{\mathrm{V}}{\mathrm{V}\mathrm{oucher}}_{\mathrm{j}}+{\upbeta}_{\mathrm{F}}{\mathrm{female}}_{\mathrm{i}}+{\upbeta}_{\mathrm{V}\mathrm{F}}{\mathrm{V}\mathrm{oucher}}_{\mathrm{j}}\times {\mathrm{female}}_{\mathrm{i}}+{\mathrm{X}}_{\mathrm{i}\mathrm{j}}+{\upvarepsilon}_{\mathrm{i}\mathrm{j}},$$

where female_i_ is a dummy variable indicating whether the student is female. The coefficient β_V_ compares eyeglasses uptake or usage in the Voucher group to that in the Prescription group, and *β*_*F*_ captures the effect of being a female student on eyeglasses uptake or usage. The coefficients on the interaction terms β_VF_give the additional effect (positive or negative) of the voucher on eyeglasses uptake or usage for female students relative to the voucher effect for male students.

In all regression models, we adjust standard errors for clustering at the school level using the cluster-corrected Huber-White estimator. All analyses were performed using Stata 16.0 (Stata Corp., Texas, USA).

## Results

### Baseline eyeglasses usage among male and female students with myopia

Table 2 shows the differences between male and female students with myopia at baseline. In general, female students have significantly higher LogMAR scores than male students, indicating worse visual acuity (Table 2, row 1, significant at the 5% level). However, caregivers of female students are four percentage points less likely to have taken girls to vision examinations prior to the study (Row 2, significant at the 10% level). Considering that less than 35% of students with myopia had been taken to vision examinations by their caregivers, four percentage points means girls are 13% less likely than boys to have received a vison exam, indicating that caregivers are seeking healthcare at lower rates for their female children.

**Table 2:** Difference between male and female children in baseline

	Female	Male	Difference	*p*-value
Variable	(1)	(2)	(1)–(2)	(1)–(2)
1. Baseline visual acuity (LogMAR)	0.646	0.622	0.024**	0.010**
	(0.007)	(0.007)		
2. Family has taken student to a vision exam (1 = yes)	0.307	0.347	−0.040*	0.055*
	(0.014)	(0.015)		
3. Student has glasses at baseline (1 = yes)	0.179	0.196	−0.017	0.337
	(0.012)	(0.013)		
4. Student can see the blackboard from his/her seat (1 = yes)	0.523	0.492	0.030	0.173
	(0.016)	(0.016)		
5. Student blinks eyes or turns head to see things clearly (1 = yes)	0.620	0.637	−0.017	0.424
	(0.015)	(0.015)		
6. Myopia knowledge index (Score 0–9)	2.903	3.175	−0.271***	0.000***
	(0.054)	(0.055)		
7. Student thinks he/she is myopic (1 = yes)	0.440	0.480	−0.040*	0.072*
	(0.015)	(0.016)		
Number of Observations	*n* = 991	*n* = 1033		
	48.96%	51.04%		

*Notes*: ***, **, and * indicate significance at the 1, 5, and 10% critical level. Standard errors are clustered at school level. Higher LogMAR values indicate worse visual acuity. Here, it means female students in our sample have worse visual acuity than male students

The results also show that female students are less aware of myopia than male students. In our sample, girls scored about 0.3 points (or about 10%) lower, on average, in myopia knowledge compared to boys (Table 2, Row 6, significant at the 1% level). Moreover, only 44% of girls recognized that they are myopic, compared to 48% of boys (Row 7, significant at the 10% level). However, there is no significant difference between girls and boys at baseline in terms of eyeglasses ownership, whether they can see the blackboard in the classroom, or whether they need to blink their eyes or turn their heads to see more clearly (Table 2, Rows 3, 4, and 5).

We next examine gender differences among different subgroups in our sample, focusing on household assets (Table 3) and left-behind child status (Table 4). Table 3 compares male and female students in the bottom 25% and the top 75% of household asset index. We find that although both subgroups show some gender differences, these differences are amplified for students in the bottom 25% of household assets. While female students in both subgroups are significantly more likely to be myopic (Table 3, row 1), only students in bottom 25% of household assets group show gendered differences in vision care prior to the study. Specifically, among students in the bottom 25% of household assets, female students are 15% percentage points less likely than male students to have been taken by their caregiver to a vision exam (Table 3, row 2, significant at 1%). Similarly, although female students in both subgroups scored lower in myopia knowledge than male students (Table 3, row 6, significant at 10 and 1%, respectively), female students in the bottom 25% of household assets are 12 percentage points less likely than male students to have realized they are myopic (Table 3, row 7, significant at 5%). In contrast, there is no statistically significant gender gap in receiving vision care or in myopia self-awareness among students in the top 75% of household assets.

**Table 3:** Difference between male and female children in baseline in different income categories

	Children from Bottom 25% Income				Children from Top 75% Income			
	Female	Male	Difference	p-value	Female	Male	Difference	*p*-value
Variable	(1)	(2)	(1)–(2)	(1)–(2)	(3)	(4)	(3)–(4)	(3)–(4)
1. Baseline visual acuity (LogMAR)	0.662	0.615	0.047**	0.029**	0.645	0.624	0.021*	0.055*
	(0.015)	(0.015)			(0.008)	(0.008)		
2. Family has taken student to a vision exam (1 = yes)	0.217	0.369	−0.152***	0.000***	0.339	0.339	0.000	0.987
	(0.027)	(0.031)			(0.017)	(0.018)		
3. Student has glasses at baseline (1 = yes)	0.146	0.199	−0.053	0.132	0.196	0.200	−0.004	0.851
	(0.024)	(0.026)			(0.014)	(0.015)		
4. Student can see the blackboard from his/her seat (1 = yes)	0.566	0.504	0.062	0.182	0.505	0.491	0.013	0.614
	(0.033)	(0.033)			(0.018)	(0.019)		
5. Student blinks eyes or turns head to see things clearly (1 = yes)	0.562	0.627	−0.065	0.154	0.639	0.632	0.006	0.799
	(0.033)	(0.032)			(0.017)	(0.018)		
6. Myopia knowledge index (Score 0–9)	2.619	2.928	−0.308*	0.062*	2.992	3.241	−0.249***	0.006***
	(0.116)	(0.117)			(0.063)	(0.065)		
7. Student thinks he/she is myopic (1 = yes)	0.336	0.453	−0.117**	0.010**	0.478	0.491	−0.013	0.619
	(0.031)	(0.032)			(0.018)	(0.019)		
Number of Observations	*n* = 226	*n* = 236			*n* = 910	*n* = 887		

*Notes*: ***, **, and * indicate significance at the 1, 5, and 10% critical level. Standard errors are clustered at school level

**Table 4:** Difference between male and female children at baseline in left-behind families and non-left-behind families

	Left-Behind Children				Non Left-Behind Children			
	Female	Male	Difference	*p*-value	Female	Male	Difference	*p*-value
Variable	(1)	(2)	(1)–(2)	(1)–(2)	(1)	(2)	(1)–(2)	(1)–(2)
1. Baseline visual acuity (LogMAR)	0.667	0.601	0.067**	0.025**	0.644	0.625	0.019*	0.058*
	(0.020)	(0.021)			(0.007)	(0.007)		
2. Family has taken student to a vision exam (1 = yes)	0.301	0.279	0.022	0.718	0.308	0.355	− 0.047**	0.034**
	(0.042)	(0.044)			(0.015)	(0.016)		
3. Student has glasses at baseline (1 = yes)	0.171	0.173	−0.002	0.963	0.180	0.198	−0.018	0.325
	(0.034)	(0.037)			(0.013)	(0.013)		
4. Student can see the blackboard from his/her seat (1 = yes)	0.520	0.442	0.078	0.243	0.523	0.498	0.025	0.294
	(0.045)	(0.049)			(0.017)	(0.017)		
5. Student blinks eyes or turns head to see things clearly (1 = yes)	0.618	0.712	−0.094	0.139	0.620	0.628	−0.008	0.721
	(0.044)	(0.045)			(0.016)	(0.016)		
6. Myopia knowledge index (Score 0–9)	2.821	3.173	−0.352	0.157	2.914	3.175	−0.260***	0.001***
	(0.160)	(0.192)			(0.058)	(0.058)		
7. Student thinks he/she is myopic, 1 = yes	0.463	0.471	−0.008	0.908	0.437	0.481	−0.044*	0.061*
	(0.045)	(0.049)			(0.016)	(0.017)		
Number of Observations	*n* = 123	*n* = 104			n = 1033	n = 991		

*Notes*: ***, **, and * indicate significance at the 1, 5, and 10% critical level. Standard errors are clustered at school level

Table 4 compares male and female students who are left-behind children (meaning that both parents have out-migrated) and non-left-behind children (meaning that at least one parent lives at home). As in Table 3, female students in both subgroups have significantly worse vision than male students (Table 1, row 1, significant at 5 and 1%, respectively). However, although we find no other significant gender differences among left-behind children, there are several significant gender differences in the non-left-behind subgroup. Specifically, caregivers of female non-left-behind children are 5% less likely to have taken their child to a vision exam than caregivers of male children in the same subgroup (Table 4, Row 2, significant at 5%). Additionally, among non-left-behind children, female students have significantly less knowledge of myopia and are significantly less likely to have recognized that they have myopia compared to their male counterparts (Table 4, Row 6 and Row 7, significant at 1% level and 5% level, respectively).

### Average impact of vouchers on student eyeglasses uptake and usage

Table 5 shows the average impacts of the intervention on eyeglasses uptake and usage for all myopic students. Columns 1 to 4 show the results for eyeglasses uptake, with columns 1 and 2 reporting uptake in the short-term (one month after vouchers were distributed) and columns 3 and 4 reporting uptake in the long-term (seven months after vouchers were distributed). Columns 5 to 8 show the estimates for eyeglasses usage, with columns 5 and 6 presenting short-term results and columns 7 and 8 presenting long-term results. Odd-numbered columns show the results using the unadjusted model (Eq. (1)), and even-numbered columns show the results using the adjusted model (Eq. (2)).

**Table 5:** Average impact of providing voucher on eyeglasses uptake and usage

	Eyeglasses Uptake				Eyeglasses Usage			
	Short term		Long term		Short term		Long term	
	(1)	(2)	(3)	(4)	(5)	(6)	(7)	(8)
	Unadjusted	Adjusted	Unadjusted	Adjusted	Unadjusted	Adjusted	Unadjusted	Adjusted
1. Voucher	0.606***	0.615***	0.447***	0.454***	0.446***	0.451***	0.246***	0.265***
	(0.022)	(0.020)	(0.024)	(0.024)	(0.025)	(0.023)	(0.027)	(0.025)
*Control variables*								
2. Age (Years)		0.011		0.020**		0.021**		−0.000
		(0.009)		(0.010)		(0.010)		(0.013)
3. Female (1 = yes)		0.009		−0.002		−0.005		0.001
		(0.016)		(0.019)		(0.018)		(0.021)
4. Boarding at school (1 = yes)		0.017		0.005		0.051*		0.007
		(0.027)		(0.034)		(0.029)		(0.032)
5. Grade four (1 = yes)		−0.009		0.004		−0.009		−0.041*
		(0.017)		(0.021)		(0.023)		(0.024)
6. Student has eyeglasses at baseline (1 = yes)		0.468***		0.302***		0.494***		0.363***
		(0.047)		(0.033)		(0.046)		(0.033)
7. Left-behind child (1 = yes)		−0.001		−0.035		−0.003		−0.047
		(0.022)		(0.030)		(0.031)		(0.032)
8. Visual acuity of worse eye (LogMAR)		0.085**		0.181***		0.086*		0.302***
		(0.042)		(0.049)		(0.049)		(0.053)
9. Father has completed high school (1 = yes)		0.003		−0.005		0.025		0.022
		(0.022)		(0.026)		(0.023)		(0.030)
10. Mother has completed high school (1 = yes)		0.014		0.005		−0.000		0.020
		(0.026)		(0.030)		(0.031)		(0.030)
11. At least one family member wears glasses (1 = yes)		0.020		0.032		0.023		0.047**
		(0.018)		(0.021)		(0.020)		(0.021)
12. Household asset index		0.004		0.013		0.003		0.009
		(0.007)		(0.008)		(0.006)		(0.008)
13. Distance from school to the county seat (km)		−0.002***		−0.001		−0.002**		0.001**
		(0.001)		(0.001)		(0.001)		(0.001)
14. Constant	0.262***	0.039	0.456***	0.075	0.227***	−0.131	0.379***	0.042
	(0.015)	(0.102)	(0.019)	(0.126)	(0.016)	(0.120)	(0.019)	(0.155)
15. Observations	1989	1980	1950	1941	1989	1980	1950	1941
16. R-squared	0.411	0.533	0.262	0.332	0.258	0.395	0.122	0.231
17. Mean in prescription group	0.248		0.427		0.210		0.345	

The results show that the eyeglasses uptake rate of the Voucher group is significantly higher than that of the Prescription group in both the short-term and the long-term. In the unadjusted model, at the time of the short-term follow-up, the average eyeglasses uptake rate among children that received only a prescription was about 24.8% (Table 5, column 1, row 17). The uptake rate among children in the Voucher group was 60.6 percentage points higher (column 1, row 1), which is nearly three times the uptake rate of the Prescription group. At the time of the long-term follow-up, the average eyeglasses uptake rate among the Prescription group was about 42.7% (column 3, row 17), while the rate among the Voucher group was 87.4%, which is 44.7 percentage points higher than the Prescription group (column 3, row 1). The adjusted model results, which control for student and family characteristics, return a similar result: the eyeglasses uptake rate in the Voucher group was 61 percentage points higher than the Prescription group at the time of the short-term follow-up (column 2, row 1) and 45 percentage points higher at the time of the long-term follow-up (column 4, row 1). These results are all significant at the 1% level.

Our results also reveal that eyeglasses usage in the Voucher group was significantly higher than the Prescription group in both the short-term and the long-term. Estimates using the unadjusted model show that in the short term, the percentage of students who use eyeglasses was 66% in the Voucher group (Table 5, column 5, row 1), which is more than three times higher than the 21% usage rate in the Prescription group (column 5, row 17). The long-term results show that the percentage of students who use eyeglasses was 59% in the Voucher group (column 7, row 17), compared to only 34.5% in the Prescription group. Results from our adjusted model also show that the Voucher increased eyeglasses usage by 45 percentage points in the short term (column 6, row 1) and 27 percentage points in the long term (column 8, row 1). These results are also significant at the 1% level.

### Heterogeneous effects on female students

This section examines the main question of interest in the paper: do information and subsidies reduce gender bias in healthcare seeking behavior among caregivers? Table 6 presents the results of our descriptive analysis examining eyeglasses uptake and usage among boys and girls in both the short-term and the long-term. The results of this descriptive analysis show that there are no statistically significant gender differences in eyeglasses uptake and usage in the short-term and the long-term among both the Prescription and Voucher groups.

**Table 6:** Difference between male and female children in eyeglasses uptake and usage at follow up surveys

	Female	Male	Difference	*P*-value
	(1)	(2)	(2)–(1)	
***Prescription Group***				
Uptake Short term (One month)	0.249	0.266	−0.017	0.544
	(0.024)	(0.024)		
Uptake Long term (Seven months)	0.440	0.463	−0.024	0.473
	(0.029)	(0.029)		
Usage Short term (One month)	0.208	0.236	−0.028	0.317
	(0.023)	(0.024)		
Usage Long term (Seven months)	0.373	0.372	0.001	0.970
	(0.027)	(0.030)		
***Voucher Group***				
Uptake Short term (One month)	0.890	0.855	0.035	0.170
	(0.019)	(0.025)		
Uptake Long term (Seven months)	0.917	0.896	0.020	0.395
	(0.018)	(0.021)		
Usage Short term (One month)	0.674	0.682	−0.008	0.813
	(0.030)	(0.033)		
Usage Long term (Seven months)	0.635	0.630	0.005	0.884
	(0.029)	(0.031)		

*Notes*: The value displayed for t-tests are the differences in the means across the groups. Standard errors are clustered at school level

We also conduct a multivariate analysis of the gendered treatment effects on eyeglasses uptake and usage in the short- and long-term. The results of this analysis are presented in Table 7. Overall, our multivariate analysis corroborates the findings of our descriptive analysis: there are no significant differences in treatment effects for male and female students, indicating that there is no greater or lesser response to the voucher based on student gender (Table 7, row 3).

**Table 7:** Heterogeneous impact of providing voucher on eyeglasses uptake and usage

	Eyeglasses Uptake				Eyeglasses Usage			
	Short term (One month)		Long term (Seven months)		Short term (One month)		Long term (Seven months)	
	(1)	(2)	(3)	(4)	(5)	(6)	(7)	(8)
	Unadjusted	Adjusted	Unadjusted	Adjusted	Unadjusted	Adjusted	Unadjusted	Adjusted
1. Voucher Group	0.581***	0.598***	0.427***	0.444***	0.440***	0.458***	0.246***	0.270***
	(0.027)	(0.025)	(0.030)	(0.030)	(0.031)	(0.028)	(0.036)	(0.035)
2. Female	−0.011	−0.000	−0.017	− 0.004	−0.014	− 0.005	0.011	0.019
	(0.027)	(0.021)	(0.032)	(0.031)	(0.027)	(0.021)	(0.031)	(0.031)
3. Voucher * Female	0.047	0.030	0.040	0.021	0.012	−0.004	−0.000	−0.018
	(0.035)	(0.032)	(0.039)	(0.039)	(0.041)	(0.038)	(0.042)	(0.042)
Baseline controls		Yes		Yes		Yes		Yes
Constant	0.268***	0.041	0.464***	0.072	0.234***	−0.122	0.374***	0.105
	(0.018)	(0.111)	(0.025)	(0.123)	(0.020)	(0.119)	(0.025)	(0.151)
Treatment Effect for Male	0.581***	0.598***	0.427***	0.444***	0.440***	0.458***	0.246***	0.270***
	(0.027)	(0.025)	(0.030)	(0.030)	(0.031)	(0.028)	(0.036)	(0.035)
Treatment Effect for Female	0.628***	0.628***	0.467***	0.466***	0.452***	0.454***	0.246***	0.252***
	(0.029)	(0.027)	(0.032)	(0.031)	(0.036)	(0.031)	(0.033)	(0.030)
Observations	1989	1980	1950	1941	1989	1980	1950	1941
R-squared	0.425	0.540	0.280	0.353	0.275	0.406	0.142	0.253
Mean in prescription Group	0.248		0.427		0.210		0.345	

*Notes*: Columns (1) to (8) show coefficients on treatment group indicators estimated by OLS. Columns (1) to (4) report estimates impact of providing voucher on eyeglasses uptake. Columns (4) to (8) report estimates impact of providing voucher on eyeglasses usage. Columns (1) (2) (5) and (6) report the short-term follow up one month after initial voucher distribution. Columns (3) (4) (7) and (8) report estimates for the long-term follow up seven months after initial voucher or prescription distribution

Standard errors clustered at school level are reported in parentheses. All regressions control for randomization strata indicators

** indicate significance at the 5% critical level

To check the robustness of our findings, we also examine the heterogenous treatment effects on girls in the bottom 25% of household assets (Additional file 2: Table 2), as these families might have greater resource constraints and may be more likely to allocate their limited resources to male children. The results show that, in fact, low-income families are more likely to redeem their voucher in the long term (row 5). Moreover, there is no significant difference in the healthcare uptake rates of girls and boys regardless their household assets (row 6).

We also examine the treatment effect for girls who are left-behind children (Additional file 3: Table 3). On the one hand, because left-behind children are left in the care of surrogate caregivers such as grandparents, left-behind girls may receive less health-related attention than boys due to time constraints and reduced parental influence. On the other hand, the baseline results shown in Table 4 suggest that girls from non-left-behind families receive vision care at lower rates compared to boys, while left-behind children show no significant gender gap in vision care. The results of our heterogeneous analysis show that left-behind children have higher eyeglasses uptake rates than non-left-behind children in both the short- and long-term (row 5). However, in both groups, there are no gender differences in uptake rates, either in the short-term or the long-term (row 6).

In short, the results from subgroup heterogenous analysis show that across different family types, rural caregivers in our sample systematically seek proactive healthcare for both girls and boys in response to the intervention. Although we observed from the baseline data that female children receive less health-related attention from their caregivers (as seen by the lower rates of vision exams among girls), when caregivers are provided with subsidized vouchers or prescription information, they do seek healthcare for their children, regardless of gender.

## Discussion

Gender translates into differential patterns of health and wellbeing for people with different cultural and social status through multiple pathways. A recently published Lancet review on gender equity, norms, and health offers a conceptual framework that summarizes the following gendered pathways to health [43] (a) gendered difference in domestic and occupational exposure; (b) gendered (risky) health behaviors; (c) gendered impacts on accessing care; (d) gender-based health systems; and (e) gendered-based health research, institutions, and data collection. Following this conceptual framework, our empirical analysis makes a specific contribution to the existing literature regarding gendered pathways to health for both (c) gendered impacts on accessing care and (d) gender-based health systems. Extant literature has shown that females are more likely to prioritize the medical needs of family members, at times at the expense of their own health [44]. Research has also shown that, partially due to the stereotyping of females as fragile or overemotional when compared to males, females’ complaints of their physical symptoms are more likely to be viewed or interpreted as over-exaggerated. As such, their health-related needs are often ignored [45].

In rural China, myopia is a health condition that is not acute and has few obvious symptoms. For this reason, it is possible that vision-related complaints of young girls may be more likely to be ignored by their caregivers. In addition, our sample area is drawn from poor areas of rural China, where the financial constraints faced by families might further limit the access of their daughters to healthcare. Finally, as discussed above [12, 24, 25, 46], rural China has traditionally valued sons over daughters, suggesting that caregivers may be more likely to allocate limited resources to more valued children (in this case, the sons in the family). Therefore, our sample area uniquely allows us to examine how rural families prioritize health-related resources (in our case, uptake and usage of correction on children’s vision problems) across girls and boys.

The study examined the gender differences in caregiver healthcare seeking behaviors in rural China using data from an in-the-field randomized controlled experiment of a program providing eyeglasses prescriptions and subsidized eyeglasses to myopic children. We first examined the gender differences in health outcomes and healthcare seeking behaviors in rural China before the intervention began. We then estimated the average impact of providing subsidized vouchers on overall healthcare uptake and usage. Finally, we explored whether families with male and female students responded differently to the healthcare intervention.

The baseline results, prior to the intervention, revealed the presence of gender differences in healthcare seeking behaviors as well as possible causes of the gender gap. We found that female students have worse vision than male students, but they are less likely to receive family attention in terms of healthcare seeking behaviors. Moreover, female students tend to be less knowledgeable about myopia and less self-aware of their myopic condition. As discussed in previous studies, these difference in health-seeking behaviors between female and male students may be caused by negligence on the part of caregivers, a lack of relevant information, or by the introverted personalities of girls [47–49].

Another possible cause of the gender difference in caregiver healthcare seeking behaviors may be financial constraints. The results of our subgroup analysis show that girls from families with low levels of household assets and girls who are non-left-behind children receive less healthcare relative to boys. In fact, it is possible – and indeed likely – that both subgroup analyses reveal that socioeconomic status is the main driver of the gender gap in healthcare. Although left-behind child status is not explicitly an indicator of socioeconomic status, studies have shown that migrant work increases a rural household’s income due to remittances from the migrating family member [50, 51]. This implies that the families of left-behind children can provide more healthcare resources to their children, as they are less financially constrained. Therefore, financial constraints may be the underlying reason for why families tend to provide less health care to their female children relative to male children.

The results from our randomized controlled experiment to provide vision care to rural students in China show that subsidies dramatically increase the uptake of healthcare compared to information alone. The results demonstrate that when Prescription information is paired with a subsidy voucher for health care (a free pair of eyeglasses), the short-term uptake rate rises dramatically from 24% in the Prescription group to 84% in the Voucher group. In the long term, the average eyeglasses uptake rate among the Prescription group is about 43%, compared to an uptake rate of 88% among the Voucher group.

The finding highlights the limited effectiveness of information interventions and confirms the work of others that have shown the importance of subsidies in expanding health care [52]. Studies have shown that awareness of a possible health risk alone is often insufficient to convince vulnerable groups to seek out care [6, 53]. This means that information-only interventions are effective only for short-term and low-cost changes in health behavior, such as dietary change and exercise compliance [54, 55]. In the case of this experiment, the ideal outcome is the uptake of eyeglasses, which is a substantial cost for family—more than a third of the average monthly income for rural households, as mentioned above. Considering that half of our sample families are from a particularly poor province, the cost of a pair of eyeglasses may be an even greater share of a family’s monthly income. Therefore, providing prescription information alone is not sufficient when such costs are involved. Instead, our results show that subsidized healthcare incentivizes caregivers to seek healthcare for their children.

Several limitations should be acknowledged when considering our results. First, we ran our analyses using data from a vision care RCT conducted in 2012 and 2013. While these data are relatively old, recent studies conducted in rural China continue to find high rates of myopia [56–58]. Additionally, to the best of our knowledge, an intervention seeking to bridge the gender gap in healthcare-seeking behaviors has not yet been implemented, suggesting that the situation in rural China largely resembles that of 2012–2013. As such, we have reason to believe our findings and conclusions regarding vision care-seeking behavior are still applicable to rural China today. Second, we are unable to rule out the possibility of omitted variable bias. Despite the scope of our data, we cannot account for all the potential characteristics that could affect healthcare seeking behaviors among caregivers.

## Conclusion

Our analysis of the differential impacts of the eyeglasses subsidy on female children contributes another layer of understanding to the gender gap in healthcare in rural China. At baseline, female children received less health-related attention from their caregivers; however, caregivers unanimously responded to both interventions (Prescription and Voucher) regardless of the gender of their children, both in the short term and in the long term. In other words, in terms of uptake of care and compliance with treatment, the voucher program benefitted female and male students equally, closing the gender gap in caregiver health-seeking behavior. More importantly, we find the same results hold true for low-income families and left-behind families.

These results suggest that the gender gap in health care among rural students is not caused by bias against girls in caregiver healthcare-seeking behaviors, although resource constraints may contribute to the gender gap. However, caregivers systematically seek proactive healthcare for both girls and boys in response to both interventions. Moreover, with subsidized healthcare removing resource constraints, healthcare uptake rates are even higher, regardless of gender.

From a policy perspective, this paper suggests that subsidized healthcare is a cost-effective way to narrow or even eliminate gender gaps in healthcare and health outcomes. One barrier for healthcare delivery in rural area is that the nearest healthcare providers are commonly located in the county seat, which can be very distant, and healthcare providers have little incentive to travel to rural schools to provide health services. Our study shows that when a subsidized healthcare is provided, a majority of caregivers will cover the travel and time cost themselves, so as to uptake healthcare in the county seat for both their boys and girls. The cost of uncorrected vision in terms of decreased quality of life and productivity so far outweigh the cost of providing subsidized vision care. Social planners could reasonably consider expanding healthcare subsidies to cover other aspects of preventative healthcare, especially those that have been shown to have preexisting gender gaps in coverage, such as vision care and mental health.

## Supplementary Information

**Additional file 1: Table 1**. Test of differential attrition at short term and long term.

**Additional file 2: Table 2**. Heterogeneous impact of providing voucher on eyeglasses uptake and usage for low income subgroups.

**Additional file 3: Table 3**. Heterogeneous impact of providing voucher on eyeglasses uptake and usage in left-behind children.

## Data Availability

The dataset used and/or analyzed during the current study are available from the corresponding author on reasonable request.

## Abbreviations

ETDRS: Early Treatment Diabetic Retinopathy Study
MAR: Minimum Angle of Resolution

## Footnotes

1 In rural China, it is common for parents to leave their children behind with a caregiver—typically the paternal grandparents—in their home communities, while the parents migrate to urban areas for work. More than 60 million children in rural China are “left-behind.”

2 Caregivers were asked to fill out a checklist of thirteen household consumption assets: automobile, truck, motor bike, tractor, farming equipment, computer, internet, television, camera, washing machine, air conditioner, water heater, gas stove, refrigerator, kitchen ventilator, and flushable toilet.

3 Measured by student responses to 9 questions about myopia, including the cause of myopia, how to cure myopia, the side effects of wearing eye glasses, etc.

4 Students with a visual acuity less than or equal to 0.5 would be considered to have a mild visual acuity loss and likely could not read the blackboard clearly in a classroom.
