# Supplementary material for: Gender equity in vision care seeking behavior among caregivers: evidence from a randomized controlled trial in rural China
Source: Int J Equity Health. 2022 Feb 19;21:26. doi: 10.1186/s12939-022-01625-4 (PMC8858451; doi:10.1186/s12939-022-01625-4)
Supplement: Supplementary file 3 — Additional file 3: Table 3. Heterogeneous impact of providing voucher on eyeglasses uptake and usage in left-behind children. [file 12939_2022_1625_MOESM3_ESM.docx]

| Appendix Table 3. Heterogeneous impact of providing voucher on eyeglasses uptake and usage in left-behind children | | | | | | | | | |
| --- | --- | --- | --- | --- | --- | --- | --- | --- | --- |
|  | Eyeglasses Uptake | | | |  | Eyeglasses Usage | | | |
|  | Short term  (One month) | | Long term  (Seven months) | |  | Short term  (One month) | | Long term  (Seven months) | |
|  | (1) | (2) | (3) | (4) |  | (5) | (6) | (7) | (8) |
|  | Unadjusted | Adjusted | Unadjusted | Adjusted |  | Unadjusted | Adjusted | Unadjusted | Adjusted |
| 1. Voucher Group | 0.575*** | 0.585*** | 0.419*** | 0.433*** |  | 0.437*** | 0.450*** | 0.249*** | 0.266*** |
|  | (0.026) | (0.023) | (0.028) | (0.026) |  | (0.030) | (0.027) | (0.032) | (0.030) |
| 2. Female (1=yes) | -0.015 | -0.011 | -0.021 | -0.014 |  | -0.026 | -0.018 | 0.005 | 0.007 |
|  | (0.024) | (0.022) | (0.026) | (0.025) |  | (0.028) | (0.025) | (0.031) | (0.029) |
| 3. Voucher*female Interaction Term | 0.058 | 0.047 | 0.046 | 0.032 |  | 0.018 | 0.004 | -0.008 | -0.021 |
|  | (0.036) | (0.032) | (0.038) | (0.036) |  | (0.041) | (0.037) | (0.045) | (0.042) |
| 4. Left-Behind Child (1=yes) | -0.071* | -0.052 | -0.113** | -0.100** |  | -0.063 | -0.047 | -0.136** | -0.120** |
|  | (0.040) | (0.036) | (0.045) | (0.043) |  | (0.046) | (0.042) | (0.053) | (0.050) |
| 5. Voucher * Left-Behind Child | 0.137** | 0.116* | 0.160** | 0.146** |  | 0.086 | 0.066 | 0.116 | 0.101 |
|  | (0.068) | (0.061) | (0.077) | (0.073) |  | (0.078) | (0.070) | (0.090) | (0.084) |
| 6. Voucher*female*Left-Behind Child Interaction Term | -0.067 | -0.075 | -0.049 | -0.064 |  | 0.005 | 0.016 | 0.069 | 0.058 |
|  | (0.076) | (0.068) | (0.084) | (0.080) |  | (0.086) | (0.078) | (0.099) | (0.093) |
|  |  |  |  |  |  |  |  |  |  |
| Baseline controls |  | YES |  | YES |  |  | YES |  | YES |
| Constant | 0.272*** | 0.044 | 0.472*** | 0.049 |  | 0.242*** | -0.163 | 0.383*** | -0.033 |
|  | (0.018) | (0.095) | (0.019) | (0.108) |  | (0.020) | (0.109) | (0.022) | (0.126) |
|  |  |  |  |  |  |  |  |  |  |
| Observations | 1,989 | 1,980 | 1,950 | 1,941 |  | 1,989 | 1,980 | 1,950 | 1,941 |
| R-squared | 0.387 | 0.517 | 0.239 | 0.324 |  | 0.211 | 0.366 | 0.071 | 0.203 |
|  |  |  |  |  |  |  |  |  |  |
| Treatment Effect for Girls | 0.633*** | 0.632*** | 0.466*** | 0.465*** |  | 0.455*** | 0.454*** | 0.241*** | 0.245*** |
|  | (0.026) | (0.023) | (0.027) | (0.026) |  | (0.029) | (0.026) | (0.032) | (0.030) |
| Treatment Effect for Left-Behind Children | 0.711*** | 0.701*** | 0.579*** | 0.579*** |  | 0.523*** | 0.516*** | 0.365*** | 0.370*** |
|  | (0.066) | (0.059) | (0.074) | (0.070) |  | (0.075) | (0.067) | (0.087) | (0.081) |
| Treatment Effect for female left-behind children | 0.508*** | 0.510*** | 0.370*** | 0.369*** |  | 0.443*** | 0.466*** | 0.318*** | 0.324*** |
|  | (0.085) | (0.076) | (0.088) | (0.088) |  | (0.096) | (0.087) | (0.110) | (0.102) |

*Notes*: Columns (1) to (8) show coefficients on treatment group indicators estimated by OLS. Columns (1) to (4) report estimates impact of providing voucher on eyeglasses uptake. Columns (4) to (8) report estimates impact of providing voucher on eyeglasses usage. Columns (1) (2) (5) and (6) report the short-term follow up one month after initial voucher distribution. Columns (3) (4) (7) and (8) report estimates for the long-term follow up seven months after initial voucher or prescription distribution.
Baseline controls including student's age, gender, boarding at school, grade, have eyeglasses at baseline, belief on eyeglasses will harm vision, whether a family member wears eyeglasses, whether parents have high school or above education, household asset index, the distance from the school to the county seat, and parental migration status. Sample sizes are less than the full sample due to observations missing at least one regressor. Standard errors clustered at school level are reported in parentheses. All regressions control for randomization strata indicators.
***, **, and * indicate significance at the 1, 5, and 10 percent critical level.
